# Fivefold higher abundance of ticks (Acari: Ixodida) on the European roe deer (*Capreolus capreolus* L.) forest than field ecotypes

**DOI:** 10.1038/s41598-021-90234-2

**Published:** 2021-05-20

**Authors:** Patrycja Opalińska, Anna Wierzbicka, Marek Asman, Grzegorz Rączka, Marcin K. Dyderski, Magdalena Nowak-Chmura

**Affiliations:** 1 Department of Game Management and Forest Protection, Poznań University of Life Sciences, Wojska Polskiego 71d, 60-625 Poznań, Poland; 2 Department of Parasitology, Faculty of Pharmaceutical Sciences in Sosnowiec, Medical University of Silesia, Katowice, Jedności 8, 41-218 Sosnowiec, Poland; 3 Department of Forest Management Planning, Poznań University of Life Sciences, Wojska Polskiego 71c, 60-625 Poznań, Poland; 4 Institute of Dendrology, Polish Academy of Sciences, Parkowa 5, 62-035 Kórnik, Poland; 5 Department of Invertebrate Zoology and Parasitology, Institute of Biology, Pedagogical University of Cracov, Podbrzezie Str. 3, 31-054 Kraków, Poland

**Keywords:** Ecology, Zoology

## Abstract

The European roe deer (*Capreolus capreolus*) is the most common deer species in Europe. The species can be a reservoir of some tick-borne diseases but it is primarily recognized for its contribution as an amplifier host. In Central Europe, two roe deer ecotypes are living in adjacent areas: field and forest. We investigated differences in tick load and species composition on these two ecotypes. We collected ticks from 160 (80 the forest ecotype and 80 the field ecotype) roe deer culled in Wielkopolska Region (West-Central Poland). The most common was *Ixodes ricinus* (n = 1610; 99%) followed by *Ixodes hexagonus* (n = 22; 1%). The dominant life stage of the ticks was female. Prevalence was higher for forest roe deer. Mean number of ticks found on the forest ecotype was almost fivefold higher than on the field ecotype (3.75 ± 0.83 vs. 0.77 ± 0.20 ticks). The mean probability of tick occurrence was threefold higher in the forest (0.915 ± 0.050) than the field ecotype (0.279 ± 0.125). The most infested body parts of roe deer from both ecotypes were the neck and the head.

## Introduction

The castor bean tick (*Ixodes ricinus* L.), the commonest human-biting tick in Europe, is most abundant in forests, especially close to animal paths^1^. In meadows and fields where it is much more drier, ticks are rare^2^. The castor bean tick is the primary vector of several bacterial, protozoan and viral zoonotic agents, e.g. tick-borne encephalitis (TBE), Lyme borreliosis (LB)^3^. The incidence of TBE has continued to rise and spread geographically^4^. *Ixodes ricinus* ticks can also transmit *Babesia* spp., *Anaplasma phagocytophilum*, *Bartonella* and *Rickettsia* species in Poland^5,6^. LB causes hundreds of thousands of new human infections worldwide annually, with numerous cases contracted in Central Europe^7,8^.

The European roe deer (*Capreolus capreolus* L.) is the most abundant cervid species occurring in Europe. It has an increasing economic, cultural, and ecological importance^9^. Roe deer can be a reservoir of several tick-borne diseases (TBD) but it is primarily recognized for its contribution as an amplifier host^10^. In some areas of Europe, e.g. Sweden, the European roe deer is the most important blood meal host for ticks^11^. The number of ticks is linked to the number of roe deer in forest environments, and as a consequence to the number of LB cases^12^ and TBE cases^13^. Therefore, the roe deer is important from a public health point of view. In Poland, Czechia and France, the field ecotype of roe deer was described^14–16^ in addition to the forest—the most widely known ecotype. Both ticks and roe deer, easily adapt to new environments, e.g. rural and suburban areas, parks and gardens^17,18^. Only a few published studies on the differences between the field and the forest ecotypes of roe deer as a host of different parasites^19–21^. The field roe deer were characterized by a higher skin parasite prevalence, but the forest roe deer had a higher infestation intensity^21^.

We aimed to assess distribution patterns of ticks in the field and the forest roe deer ecotypes. We hypothesized that (1) the field roe deer have higher ticks prevalence; (2) the forest roe deer have a higher infestation intensity, and (3) there are differences between the field and the forest ecotype of roe deer in body parts infestation and prevalence by ticks**.**

## Results

Altogether, we collected 1632 individual ticks from 77 out of 160 examined roe deer individuals, 64 males and 13 females were infested. The most common was *Ixodes ricinus* L. (1610 individuals) followed, by *Ixodes hexagonus* Leach 1815 (22 individuals), every time when *I. hexagonus* was observed it was mixed infestation with I*.* *ricinus*. *I.* *hexagonus* individuals were 1.3 percent of all collected ticks so we excluded them in further calculations. On roe deer females (examined in winter) we found 31 ticks (1.9%), thus we analysed patterns of *I.* *ricinus* occurrence only on male roe deer (examined in spring). From 1579 individuals of ticks, only 4 were larvae (1 ind. in the forest and 3 ind. in the field ecotypes), equaling 0.03 percent thus we also omitted them in analyses.

**Figure 1:**
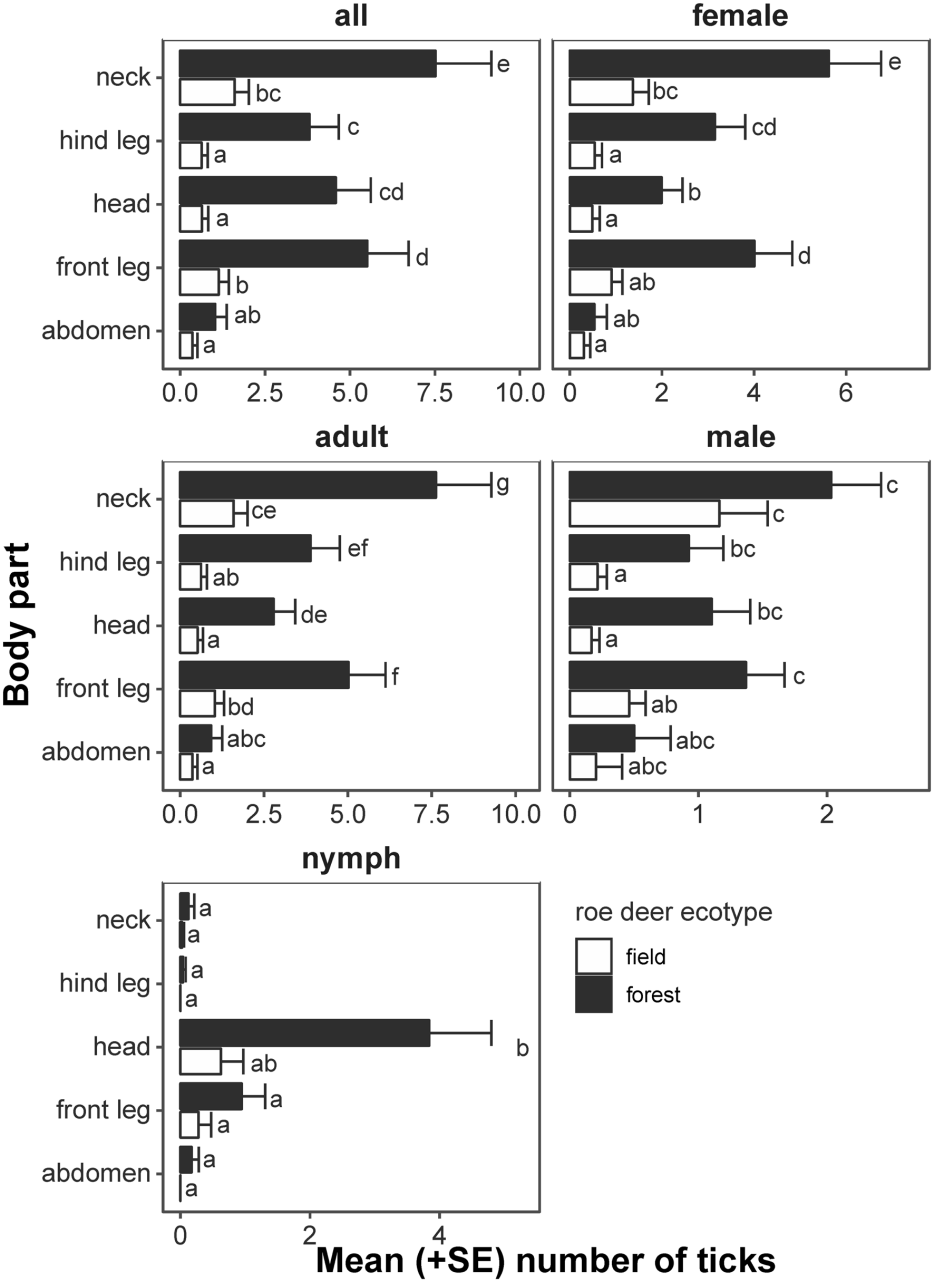
Mean (+ SD) tick number per body part and the European roe deer ecotype, predicted using GLMMs assuming zero-inflated Poisson distribution (Table 2). Variants denoted by the same letter did not differ at *p* = 0.05, according to Tukey post hoc test. The plot has been generated using R software^36^.

Among 1575 individuals of castor bean ticks found on roe deer males in spring, we 449 found on the field ecotype of roe deer and 1126 on the forest ecotype (see descriptive statistics in Table 1). Prevalence was higher for the forest roe deer (100% vs. 60%) and also intensity of infestation (the forest ecotype 28.15 ± 19.79 ind., the field ecotype 18.71 ± 13.93 ind.). The abundance for the forest ecotype was the same as intensity of infestation and for the field ecotype it was lower—11.23 ± 13.38 ind. We found that regardless of life stage examined, the forest ecotype of roe deer hosted higher number of ticks than the field ecotype (Table 2, Fig. 1). On average, the mean number of ticks found on the forest ecotype was almost fivefold higher than on the field ecotype (3.75 ± 0.83 vs. 0.77 ± 0.20 ticks). Lower differences between roe deer ecotypes were found in case of nymphs and male ticks, which were less frequent. However, in case of male ticks on neck, despite *p* > 0.05 between variants post hoc tests, the difference was significant: the forest roe deer ecotype hosted almost twice more ticks than the field ecotype (2.03 ± 0.39 vs. 1.16 ± 0.37). In case of nymphs occupying head, the difference was almost six fold (3.84 ± 0.96 vs. 0.62 ± 0.35). We also find differences among examined body parts: we found the most of ticks on neck, head and legs, while the least—on the abdomen.

**Table 1:** Descriptive statistics and N:M:F ratio regarding number of ticks at different stages and sex collected from 80 European roe deer males representing two ecotypes: the field and the forest.

Roe deer ecotype		Nymph (ind.)	Adult		Total (ind.)	N:F:M ratio
			Female (ind.)	Male (ind.)		
Field ecotype	Min	0	0	0	0	0.13:1:0.39
	Max	5	43	16	62	
	Mean ± SD	0.97 ± 1.48	7.35 ± 9.59	2.90 ± 4.09	11.23 ± 14.16	
	Median	0	5	1	6.5	
	Total	39	294	116	449	
Forest ecotype	Min	0	1	0	2	0.18:1:0.31
	Max	32	50	22	95	
	Mean ± SD	3.35 ± 6.39	18.90 ± 12.87	18.90 ± 12.87	28.15 ± 20.76	
	Median	0	16	5	25	
	Total	134	756	236	1126	
Total	Min	0	0	0	0	0.16:1:0.335
	Max	32	50	22	95	
	Mean ± SD	2.16 ± 4.76	13.12 ± 12.69	4.40 ± 4.69	19.69 ± 19.60	
	Median	0	10	3	14.5	
	Grand total	173	1050	352	1575	

N:M:F ratio was calculated as ticks’ number of nymphs and males accruing per 1 female.

**Table 2:** ANOVA of GLMMs explaining infestation abundance (assumed zero-inflated Poisson distribution).

Response	Variable	*Χ*^*2*^	*df*	*p*	Model parameters	Random effects
All ticks	Roe deer ecotype	24.5119	1	< 0.0001	AIC = 1813.4	Variance = 1.77
	Roe deer ecotype × body Part	9.2963	4	0.0541	AIC_0_ = 2139.6	SD = 1.33
	Body part	172.2561	4	< 0.0001		
Females	Roe deer ecotype	23.2019	1	< 0.0001	AIC = 1453.5	Variance = 1.43
	Roe deer ecotype × body part	5.0384	4	0.2834	AIC_0_ = 1738.7	SD = 1.199
	Body part	134.4630	4	< 0.0001		
Adults	Roe deer ecotype	23.8370	1	< 0.0001	AIC = 1696.8	Variance = 1.73
	Roe deer ecotype × body part	5.1391	4	0.2733	AIC_0_ = 2069.3	SD = 1.31
	Body part	217.0682	4	< 0.0001		
Males	Roe deer ecotype	15.0250	1	0.0001	AIC = 913.3	Variance = 1.43
	Roe deer ecotype × body part	11.2970	4	0.0234	AIC_0_ = 1018.8	SD = 1.199
	Body part	48.5230	4	< 0.0001		
Nymphs	Roe deer ecotype	10.0644	3	0.0180	AIC = 432.2	Variance = 1.20
	Roe deer ecotype × body part	1.0776	4	0.8978	AIC_0_ = 1738.7	SD = 1.09
	Body part	70.3049	6	< 0.0001		

AIC—akaike information criterion, AIC_0_—AIC of null model (intercept only) < random effects cover dependence of observations among particular roe deer individual, *Χ*^2^—test statistic, *df*—degrees of freedom, *p*—*p* value.

**Figure 2:**
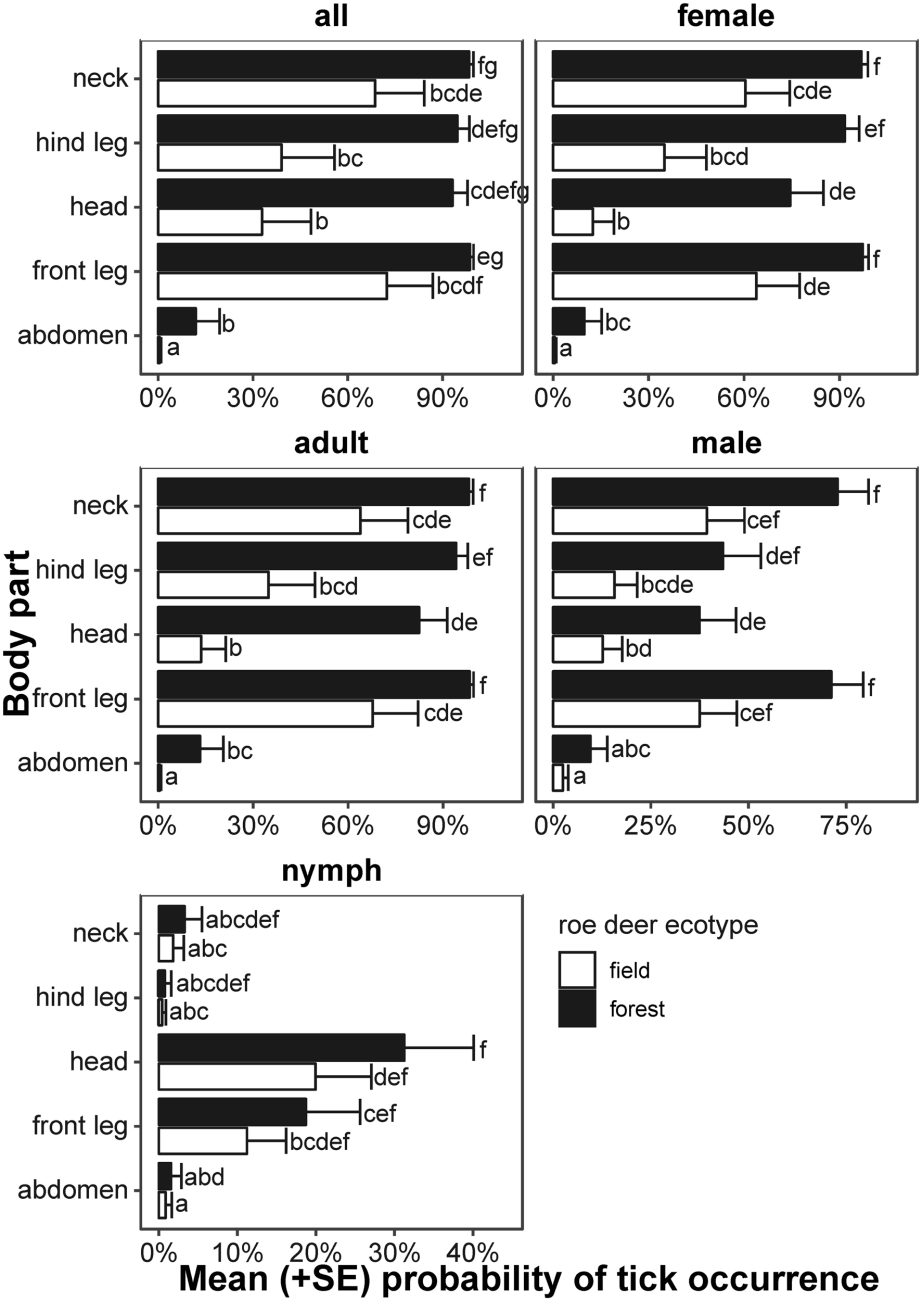
Mean (+ SD) probability of tick occurrence per body part and the European roe deer ecotype, predicted using GLMMs assuming binomial distribution (Table 3). Variants denoted by the same letter did not differ at *p* = 0.05, according to Tukey post hoc test. The plot has been generated using R software^36^.

We found higher prevalence of ticks in case of the forest ecotype of roe deer than the field ecotype (Table 3, Fig. 2). In case of all life stages pooled together, mean probability of tick occurrence was threefold higher in the forest (0.915 ± 0.050) than the field ecotype (0.279 ± 0.125). We found similar pattern in case of all life stages. Even in case of nymphs, where difference between roe deer ecotype had *p* = 0.22, we found almost twice higher probability of tick occurrence in the forest (0.051 ± 0.023) than the field ecotype (0.028 ± 0.01). In case of the forest ecotype probability of occurrence did not differ among body parts (with an exception of abdomen, where always was lower than 0.15). In contrast, examining the field ecotype we found lower probability of tick occurrence in head and hind leg, in comparison with front leg and neck. Also, we found the lowest probability of tick occurrence in abdomen (always lower than 0.025).

**Table 3:** ANOVA of GLMMs explaining infestation prevalence (assumed binomial distribution).

Response	Variable	*Χ*^*2*^	*df*	*p*	Model parameters	Random effects
All ticks	Roe deer ecotype	12.9130	1	0.0003	AIC = 342.2	Variance = 10.57
	Body part	43.3690	4	< 0.0001	AIC_0_ = 482.2	SD = 3.25
Adults	Roe deer ecotype	16.6660	1	< 0.0001	AIC = 347.1	Variance = 8.11
	Body part	55.7240	4	< 0.0001	AIC_0_ = 493.6	SD = 2.85
Females	Roe deer ecotype	16.9910	1	< 0.0001	AIC = 359.1	Variance = 6.27
	Body part	61.6570	4	< 0.0001	AIC_0_ = 509.4	SD = 2.50
Males	Roe deer ecotype	9.5352	1	0.0020	AIC = 445.4	Variance = 2.36
	Body part	45.3061	4	< 0.0001	AIC_0_ = 505.1	SD = 1.52
Nymphs	Roe deer ecotype	1.5059	1	0.2198	AIC = 247.6	Variance = 1.70
	Body part	33.3816	4	< 0.0001	AIC_0_ = 299.4	SD = 1.30

AIC—akaike information criterion, AIC_0_—AIC of null model (intercept only) < random effects cover dependence of observations among particular roe deer individual, *Χ*^2^—test statistic, *df*—degrees of freedom, *p*—*p* value.

Tables 4 and 5 show the correlation between tick life stages on one body part and the burden with other body parts. Correlation between females, males and total ticks number and body parts is higher for the field ecotype. Pattern of the burden of ticks is similar on all body parts; if the head is infested other body parts are also infested. For the forest ecotype, the correlation between the ticks life stage and infestation of body parts is not so strong. When looking at the total tick number, head burden is correlated with all other body parts, the strongest correlation is between the head and front legs.

**Table 4:** Correlations (Kendall’s tau) between numbers of tick life stages/tick sex on one body part, with tick numbers of the same life stage/sex on other body parts on European roe deer from the forest ecotype.

	Head	Neck	Front legs	Hind legs
Neck—Nymph	0.213			
Front legs—Nymph	0.360**	0.244*		
Hind legs—Nymph	− 0.045	− 0.045	− 0.093	
Abdomen—Nymph	0.143	− 0.065	0.066	− 0.037
Neck—Female	0.281*			
Front legs—Female	0.356**	0.244*		
Hind legs—Females	0.277*	0.172	0.359**	
Abdomen—Female	0.010	0.092	0.218*	0.114
Neck—Male	0.109			
Front legs—Male	0.230*	0.184		
Hind legs—Male	0.054	0.040	0.143	
Abdomen—Male	0.114	0.154	0.380***	0.059
Neck—Ticks	0.333**			
Front legs—Ticks	0.370***	0.277*		
Hind legs—Ticks	0.311**	0.122	0.395***	
Abdomen—Ticks	0.224*	0.232*	0.263*	0.148

*p ≤ 0.05; ** ≤ 0.01; *** ≤ 0.001.

**Table 5:** Correlations (Kendall’s tau) between numbers of tick life stages/tick sex on one body part, with tick numbers of the same life stage/sex on other body parts on European roe deer from the field ecotype.

	Head	Neck	Front legs	Hind legs
Neck—Nymph	− 0.103			
Front legs—Nymph	0.190	0.338**		
Hind legs—Nymph	No	No	No	
Abdomen—Nymph	No	No	No	No
Neck—Female	0.570***			
Front legs—Female	0.440***	0.646***		
Hind legs—Females	0.360**	0.614***	0.710***	
Abdomen—Female	0.324**	0.253*	0.449***	0.382***
Neck—Male	0.732***			
Front legs—Male	0.342**	0.481***		
Hind legs—Male	0.488***	0.407***	0.387***	
Abdomen—Male	0.333**	0.192	0.180	0.020
Neck—Ticks	0.616***			
Front legs—Ticks	0.534***	0.663***		
Hind legs—Ticks	0.453***	0.640***	0.647***	
Abdomen—Ticks	0.368***	0.311**	0.410***	0.347**

*p ≤ 0.05; ** ≤ 0.01; *** ≤ 0.001; no—no data.

Nymphs were more common in the forest, more abundant on the head of the forest roe deer than females but fewer on other body parts. Female to male ratio was more favorable for males in field ecosystem.

## Discussion

Our study limitations, namely almost no ticks on female European roe deer and no ticks on the animals back, was caused by legal restrictions—Hunting Law Act and the fact that culled animals belong to the leaseholder. We could examine the animals due to the good will of local hunters but the animals were later sold, so the quality of meat was more important for the hunters than our research, and the animals were disemboweled before tick collection. Due to these circumstances we couldn’t follow the Kiffner et al.^22,23^ protocol completely, but still we can compare some of our findings. Due to the reasons listed above, we couldn’t follow the protocol of Carpi et al.^13^ and cut the lower legs, it could explain why we found such a small number of tick larvae.

Different researchers used different protocols to investigate the number of ticks attached to roe deer: some used water to wash out all ticks from the whole animal skin^24,25^, some took only legs^13^, some picked ticks from the most infected parts^26–28^. Our protocol was closest to Kiffner et al.^22,23^ and comparing our results with this research is most appropriate in our opinion.

The most common tick species on European roe deer from the Wielkopolska Region was *Ixodes ricinus,* which is in line with studies from Poland^26,27^, Germany [e.g. ^28^.], Sweden^24^, Serbia^29^, Spain [e.g. ^25,30^.] and other European countries. The second species was *I.* *hexagonus* rarely found on roe deer^24,31^.

The mean tick burden per roe deer (19.69 ± 18.82 ind. in general, 28.15 ± 19.79 ind. in the forest and 11.23 ± 13.39 in the field ecosystem) was much lower than from Germany—64.49 ± 10.62 ind.^22^, in a new study from Germany the average infestation rate was 26.7 (SD = 69.5)^32^ and Spain—43.2 ± 49.85 ind.^25^. Even the maximum number of ticks on one roe deer (87 ind.) was much lower than in Germany (270 ind.). It can be caused by general circumstances—low humidity, research areas have low annual rainfall (see Material and Methods) or is a consequence of study limitations as mentioned above. Ticks are sensitive to air humidity changes^2^.

The most common life stage was female, this finding is in line with Adamska^26^, Michalik et al.^27^, Vasquez et al.^25^ but contrary to results obtained by Kiffner et al.^22^ where nymphs were most abundant. The most infected body part was the neck followed by the head, which is in line with Kiffner et al.^23^ findings. The highest density of nymphs and highest nymph to female ratio was found on the head, the same as in Germany^23^.

When it comes to different ecosystems and two different European roe deer ecotypes, the forest ecotype was more infested, all animals were infested and the intensity of infestation was higher. It is not surprising due to fact that the forest is a primeval site of *I.* *ricinus*^2^. Interesting is the fact that in both ecosystems studied ticks preferred the same body parts, especially the neck and the head. Correlation between infestation of the head and other body parts was much stronger when for the field ecotype of European roe deer.

European roe deer living in forests have fivefold higher number of ticks and threefold higher probability of tick infestation than ones living on fields. However, still 60% of field roe deer ecotype carried at least one tick. This finding is important from a public health point of view. European roe deer living on fields spread the ticks to areas in close vicinity to human settlements. There was no difference in the pattern of infestation between different roe deer ecotypes—ticks prefer the neck and the head. Thus, the conclusion of Kiffner et al.^23^ to limit tick collection to these body parts is also correct for roe deer from other ecosystems than forest.

## Methods

**Figure 3:**
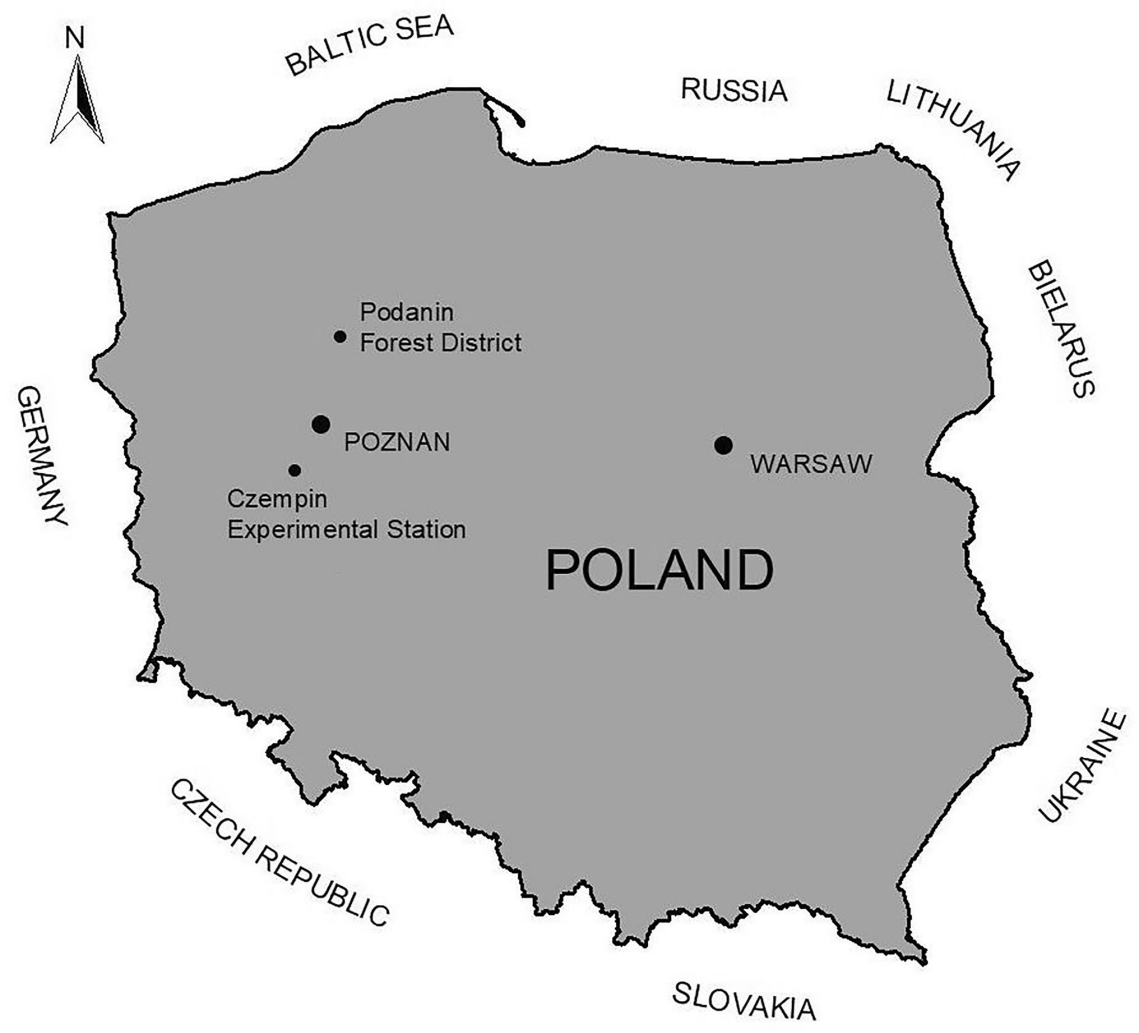
Study area at the background of Poland borders, with study site where forest (Podanin Forest District) and field (Czempiń Experimental Station) ecotypes were harvested.

We sampled European roe deer from the Wielkopolska Region (Central-West Poland). The species can live in different landscapes, in Central Europe researchers have determined two ecotypes: (1) roe deer living only in forest and (2) roe deer living only on fields, never entered forests. This two groups have different behavior and biometric features^15,18^. First we determined where the forest and the field ecotypes of European roe deer were existing and are not mixing on a daily basis. Based on literature and our earlier field observations, we chose the Podanin Forest District for the forest ecotype and Czempiń Experimental Station for the field ecotype. The field ecotype was confirmed on this area during telemetric observations between 2009 and 2012 and the population was described in details by Kamieniarz^18^ (Fig. 3). Both places have a special way (different than for rest of a country) of managing game animals called Game Breeding Centre. All management is due to welfare of animals and to increase they number.

The Podanin Forest District (18,360 ha) forest sites (according to Polish forest typology) are fresh: coniferous and mixed broadleaf-coniferous (in total 93%). Scots pine (*Pinus sylvestris* L.) trees cover 83% of the forest area^33^. The mean annual temperature is 8.0 °C, and the mean annual precipitation sum is about 550 mm.

Czempiń Experimental Station belongs to the Polish Hunting Association, it is located in the center of the Wielkopolska Region. The area is about 13,000 ha, mostly arable lands, no big forest stands nearby. The mean annual temperature is 8.4 °C and the mean annual precipitation is 478 mm^34^.

Ticks were collected from roe deer hunted from April 2015 to January 2017. The animals were hunted according to the Annual Hunting Plan and Polish Law. The hunting season for roe deer males (bucks) is from the 11th of May to the 30th of September and for roe deer females from the 1st of October to the 15th of January^35^. We collected ticks from 160 deer carcasses: 80 the forest ecotype (40 males and 40 females) and 80 the field ecotype (40 males and 40 females).

We followed the protocol developed by Kiffner et al.^22,23^ when collecting ticks. The European roe deer carcasses were disemboweled by the hunters and stored in cooling chambers at 2–8 °C until examination. Within 1–2 h after the roe deer individuals had been bagged, each carcass was examined by one observer wearing latex gloves. The carcass was divided into 5 distinct parts according to Kiffner et al.^22^. The backs were disclosed because during disemboweling, the animals were put on their backs and ticks were mechanically removed. The roe deer skin was systematically inspected and palpated to detect all ticks. All ticks were removed from each body part with forceps and then placed in plastic tubes with 70 percent ethanol. In the laboratory, the specimens were determined under the stereoscope microscope (Nikon SMZ1000, Japan), the species level and the stage of development using identification keys^2^, subsequently the ticks were placed in the plastic tubes with the ethanol and stored at − 20 °C.

The estimates of European roe deer infestation with ticks were done using the following parasitological parameters: the prevalence of infestation—a percentage of hosts carrying at least one tick; the abundance of infestation—a mean number of ticks per host and the mean intensity of infestation—the average number of ticks per tick-infested animal^27^. We calculated nymph, females and males ratio, using number of females as reference value (1).

We conducted statistical analyses using R software (The R Foundation for Statistical Computing Platform; Vienna, Austria, version 3.5.3)^36^. All the mean values are followed by ± SD. To assess the differences between European roe deer ecotypes and body parts in the infestation and prevalence of ticks, we developed generalized linear mixed-effects models (GLMMs), accounting for random effects connected with roe deer individual examined. Including random intercept for roe deer individual accounts for observations dependency connected with particular animal. In case of abundance we assumed zero-inflated Poisson distribution of dependent variable. We developed hurdle models, i.e. models predicting two parts of variable: zero-inflation (assumed as binomial-distributed probability of presence) and count (i.e. Poisson distributed predicted number of individuals). GLMMs with zero-inflated Poisson distribution assumed that both zero-inflation and count parts of model depends on roe deer ecotype, body part, and their interaction. We developed these models using glmmTMB package^37^. To assess impact of these variables on prevalence of infestation, expressed as probability of tick presence, we developed GLMMs assuming binomial distribution of dependent variable, with logit linking function. Similarly, we assumed dependence on roe deer ecotype, body part, and their interaction. Models were developed using lme4 and lmerTest packages^38,39^. In case of all GLMMs we assessed effect of studied variables in final models, assessing model performance using Akaike’s Information Criterion (AIC): we selected variables to reach the lowest AIC of the model. We also stated AIC_0_—AIC of null model (intercept-only), to show how our final models improved prediction in relation to bare the mean value. We assessed effects of roe deer ecotype and body part performing ANOVA of developed GLMMs and then—Tukey *posteriori* tests, implemented in emmeans package^40^, adjusting multiple comparison by studentized range distribution with the number of the means in the family.

We followed the protocol from Kiffner et al.^22^ and calculated Kendall’s S correlation between tick life stage abundance on one body part and life stage abundance on other body parts and the total number of this life stage on the entire roe deer carcass. We are conscious that statistical power of Kendall's correlation test is lower than parametric tests, as comparison is based on ranks. However, we decided to repeat methods used by Kiffner et al.^22^ instead GLMMs assuming zero-inflated Poisson distribution to maintain comparability of our results with previous studies.

## Data Availability

Correspondence and requests for materials should be addressed to A.W.
